# Applications of seaweed extracts in Australian agriculture: past, present and future

**DOI:** 10.1007/s10811-015-0574-9

**Published:** 2015-04-14

**Authors:** Tony Arioli, Scott W. Mattner, Pia C. Winberg

**Affiliations:** 1 Seasol International, Bayswater, Australia; 2 Australian National University, Canberra, Australia; 3 Victorian Strawberry Industry Certification Authority, Toolangi, Australia; 4 La Trobe University, Bundoora, Australia; 5 University of Wollongong Shoalhaven Campus, Nowra, Australia

**Keywords:** Biostimulants, Macroalgae, Phenomics, Crops, Pathogens

## Abstract

A rapidly growing world population has highlighted the need to significantly increase food production in the context of a world with accelerating soil and water shortages as well as climatic stressors. This situation has generated new interest in the application of liquid seaweed extracts because of their potent plant growth-enhancing properties through metabolic benefits, triggering disease response pathways and increasing stress tolerance. The basis for these benefits is complex and poorly understood. Liquid seaweed extracts are complex and have been demonstrated to possess novel mechanisms for increasing crop productivity. The benefits of seaweed extracts to crops have previously been reviewed in the context of the northern hemisphere, but not in the context of Australia, its crops and unique stressors. This review considers the application of seaweed extracts in Australian agriculture by (i) introducing the history of the Australian liquid seaweed extract industry and (ii) focusing on evidence of Australian research related to seaweed extract composition, plant growth properties during plant establishment, pathogenic disease and new approaches to phenotyping the biological efficacy of seaweed extracts. This type of research is essential for future Australian agriculture to develop effective strategies for the use of liquid seaweed extracts.

## Scope of review

The purpose of this article is to review the application of seaweed extracts in Australian agriculture by highlighting new and emerging Australian research related to (i) the status of chemical characterisation of seaweed extracts, (ii) the application of seaweed extracts to improve plant establishment and to suppress three plant pathogens, i.e. *Albugo candida* (the cause of white blister), *Plasmodiophora brassicae* (which causes clubroot) and *Sclerotina minor* (the cause of lettuce drop and other diseases) and (iii) the development of new methods for phenotyping the bioefficacy of liquid seaweed extracts.

## Introduction

**Fig. 1:**
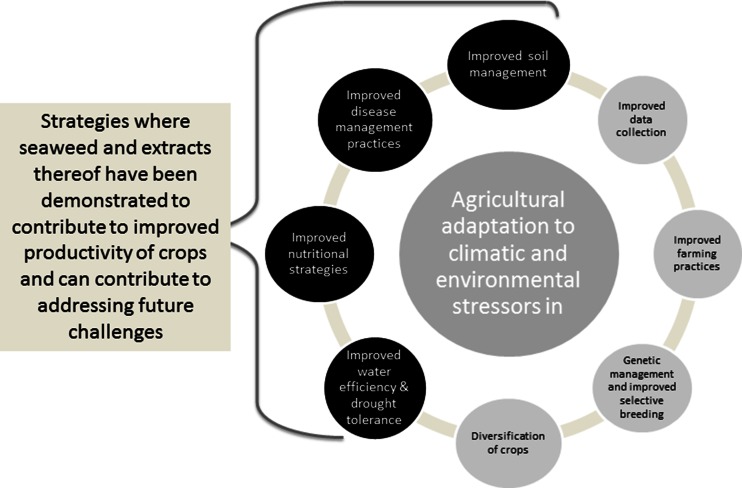
A suite of strategies that will contribute to adaptation of crop production in Australia in light of increasing climatic and environmental pressures that are reducing productivity

Farming in Australia is particularly challenging, and the shifting environment and climate is a major concern. Australian farmers frequently encounter devastating climatic events such as heatwaves, floods, drought, frost, cold and water limitations. In recent times, heatwaves have become more extreme, they are hotter for longer, commence earlier in the season and occur more often. In 2013, heatwaves surpassed all previous Australian records (Steffen et al 2014). This type of extreme climatic volatility is now challenging the historical drivers for agricultural crop gain, improved crop genetics and improved crop management (Edgerton 2009). Crops have not been bred for the extreme climate conditions, and management practices are being challenged beyond their infrastructure and capacity. Consequently, there is an urgent need to find new ways to increase agricultural resistance to stressors and boost productivity to supply the population’s unprecedented demand for food. As a regular component of agricultural programmes, seaweed extracts represent a major opportunity to significantly enhance crop gain and resistance to stress and disease in the future (Fig. 1).

The use of seaweeds has a long history dating back to Roman times (Henderson 2004). More recently, the scientific benefits of applying seaweed extracts in agriculture have been extensively reported and well-reviewed in peer-reviewed scientific publications and more broadly in the plant biostimulant literature (Khan et al 2009; Craigie 2011; Du Jardin 2012; Calvo et al 2014). However, despite the growing evidence for unique, highly specific and complex functionality of diverse molecules in seaweed extracts, their complex modes of action remain elusive. Nevertheless, seaweed extracts are already delivering improved agricultural productivity, and a greater understanding of their biological modes of action will further enhance productivity in the future. The current status and context for seaweed applications in Australian agriculture are presented here.

## A diversity of seaweed extracts

**Fig. 2:**
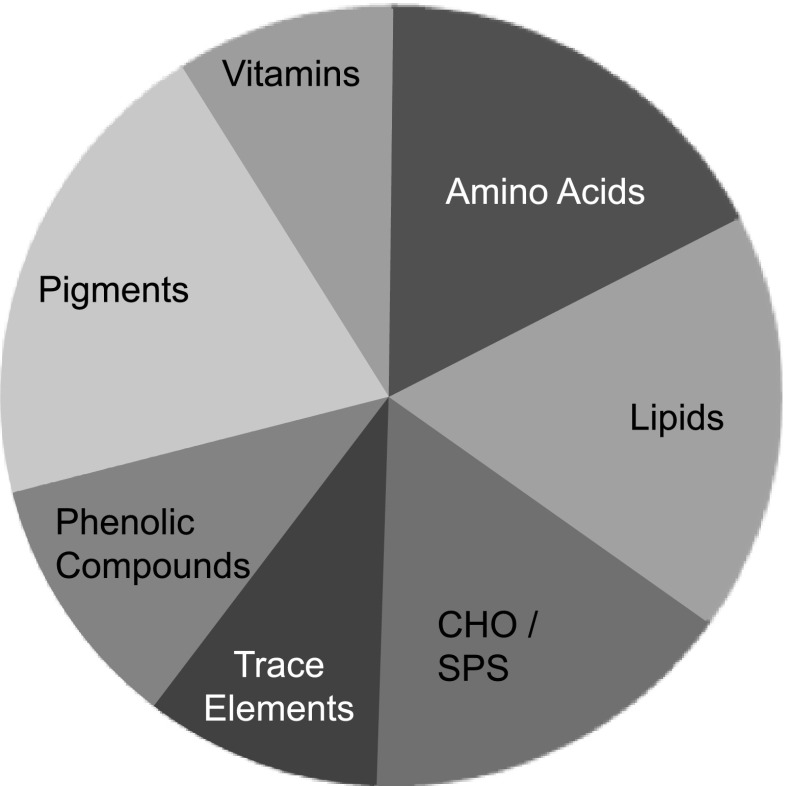
Adapted from Winberg et al. (2014). The distribution of research effort across seaweed metabolites, phycological research papers based on a Google Scholar database search of *n* = 23,800

Liquid seaweed extracts are processed from seaweed biomass using different manufacturing systems such as alkaline or acid hydrolysis or cellular disruption under pressure or fermentation. Thus, extracts comprise diverse fractions of very diverse molecules and are heterogeneous in nature. In the literature, the emphasis of research has been distributed across seven crude categories (Fig. 2). In essence, the raw extracts, apart from any processing additives, are a liquid fraction concentrate reflecting the complex chemical composition of the seaweed plants. Initially, liquid seaweed extracts were regarded as a tonic because of their medicinal-like properties for enhancing plant growth. However, the current status reflects an increasingly sophisticated knowledge of metabolic compounds with direct effects on plant metabolism or indirect effects through the soil microbiome or by interactions with pathogens. Broadly, seaweed extracts are regarded as plant biostimulants, and a precise definition for biostimulants in agriculture has been proposed by the industry for consideration by the EU regulatory authority (Du Jardin 2012).

This review focuses upon extracts manufactured entirely from seaweed (as opposed to formulations) and some of the mechanisms that provide plant and soil benefits. Diluted and blended products made from seaweed extract and synthetic fertiliser, or other plant stimulants, can create confusion regarding their true modes of action and are not covered.

## The Australian seaweed extract industry

Seaweeds collected from the beach have been exploited for centuries and are still used today, for their ability to improve soil nutrition and structure through composting and their apparent and documented plant growth stimulant properties. However, it was the development of a process to liquefy seaweed biomass that revolutionised the use of seaweeds in agriculture (Milton 1952). The availability of liquid seaweed extracts and their ease of use pioneered the creation of a commercial seaweed extract industry in England around the 1950s and enabled their widespread use in agriculture (Booth 1969; Craigie 2011).

The use of liquid seaweed extracts in Australia began around the 1970s (Abetz 1980). The first Australian company (Tasbond Pty Ltd.) to start the manufacture of a liquid organic seaweed extract was established by a group of scientists, and the company was formally registered in 1970. In 1974, the company’s first commercial production of the liquid organic seaweed known as Seasol™ was in Tasmania (Australia). Then, manufacture was solely based on a local Tasmanian giant bull kelp (*Durvillaea potatorum*), and the seaweed biomass was hydrolysed using an alkaline process. The bull kelp was sourced from kelpers (harvestors) that collected the storm-cast seaweed from the pristine beaches of King Island (Australia). Tasbond Pty Ltd. was later purchased and since 1984 traded as Seasol International Pty Ltd. It has successfully pioneered the use of seaweed products in the Australian commercial and home garden segments and exported the liquid organic seaweed product for over 20 years.

Currently in Australia, many types of seaweed extracts can be purchased for commercial agriculture and home gardening. Some of these extracts are manufactured in Australia, for example, Fair Dinkum Fertilizers™ and Natrakelp™, while others are imported brands such as Acadian™ and Kelpak™. Overall, a significant level of diversity exists in liquid seaweed products available in Australia due to the different processes used in manufacture (alkaline hydrolysis, fermentation, and pressure disruption) and the range of seaweed species used in production (*Ascophyllum nodosum*, *Durvillaea potatorum* and *Ecklonia maxima*). However, there are broader ranges of seaweed extracts produced globally (Craigie 2011; Khan et al 2009). The global production estimate of seaweed biomass for soil and plant applications is well over 550,000 tonnes per annum (Nayar and Bott 2014).

## What are the benefits of using seaweed extracts in Australian agriculture?

**Fig. 3:**
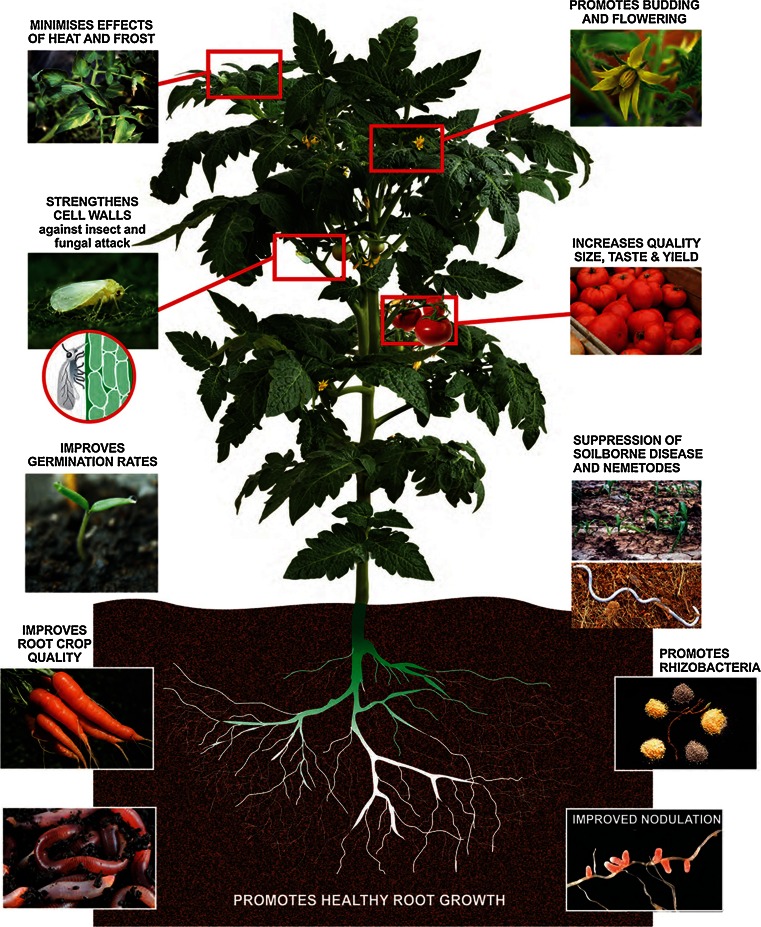
General summary of the plant and soil benefits reported upon application of liquid seaweed extracts. (Reproduced with permission from Seasol International.)

Seaweed extracts have been reported to assist plants in many ways (reviewed in Khan et al 2009; Craigie 2011; Du Jardin 2012; Calvo et al 2014). The benefits are outlined in Fig. 3 and included enhanced crop yield, improved root structures, improved plant development like flowering and leaf development and fruit set, and enhanced ability to tolerate plant disease and climatic stresses such as cold or drought. There are also benefits that relate to improved soil structure, soil water holding capacity and improved soil microbiology. However, the modes of action for these benefits are not well understood, despite increasingly sophisticated research reported in quality scientific publications. Common research findings have focused on:

1. Many types of plant growth regulators that have been identified in seaweed extracts, such as auxins, cytokinins, ethylene, gibberellins, abscisic acid and more (Tay et al 1985, 1987; Crouch et al 1992; Stirk and van Staden 1997; Khan et al 2009; Kurepin et al 2014). In early work, liquid seaweed extracts were characterised by plant callus-inducing assays, and the responses were equated to growth regulators (i.e. specific plant hormone) equivalents (Craigie 2011). In seaweeds, the first definitive discovery of cytokinins (zeatin, zeatin-riboside, dihydro-zeatin, dihydro-zeatin-riboside) was found in *Durvillaea potatorum*, using the Seasol™ liquid seaweed extract that was sold commercially in Australia (Tay et al 1985). Recently, Stirk et al. (2014) reported the presence of brassinosteroids (along with gibberellins and abscisic acid) in a South African kelp extract (Kelpak™—manufactured from *E. maxima*).
2. Quaternary ammonium molecules, such as betaines and proline, that buffer against major osmotic changes were reported by Blunden et al 1986a; Blunden et al 1986b; Wani et al 2013; Karabudak et al 2014. These osmoprotectants have an important role in plant stress and importantly have been observed to accumulate during increased stress tolerance (Calvo et al 2014). Betaines have been reported in several brown algae genera such as *Ascophyllum*, *Fucus*, *Laminaria* (Craigie 2011).
3. Alginate and diverse polysaccharides, some sulphated, have been characterised that (i) stimulate root growth both directly and indirectly in association with microbes (Xu et al 2003; Khan et al. 2012; González et al 2013), (ii) trigger the plant’s defence mechanisms (Subramanian et al 2011) and (iii) induce plant genes involved in pathogenesis-related defence (Vera et al 2011).
4. Minerals and trace elements that enhance nutrition or have a critical role in plant development, along with lipid-based molecules such as sterols were reported by Mancuso et al. (2006) and Rayirath et al. (2009).

In addition, seaweed extracts have many other molecules that are typically found in plants, which are not characterised, but might also contribute to the efficacy of various seaweed extracts. This is consistent with genomic and cell biology bioassay studies using seaweed extracts (Rayirath et al 2009; Khan et al 2011). Bioinformatics studies have uncovered hundreds of plant genes that respond when plants are treated with seaweed extracts (Nair et al 2012; Jannin et al 2013).

In Australia, seaweed extracts are likely to attract increased scientific attention in the agricultural field, especially as they develop upon their proven usage in Australia and other countries. However, for agriculture to fully exploit the biological benefits in seaweed extracts, a major cross-disciplined research effort will be needed to elucidate their complex modes of action and applications on diverse crops and in different production environments. In addition, we need to recognise that seaweed extracts are inherently different as they are derived from different sources and extraction processes and have particular extract stability properties (Stirk et al 2014). Furthermore, their capacity to elicit plant responses also depends in part upon the application usage rates, application frequency and the timing of applications in relation to plant development life cycle. Therefore, new translational research is needed for determining the appropriate times and plant stages for their application and for defining the optimal dosages required to maximise both farm productivity and economic benefits. For Australian agriculture to benefit optimally from the use of seaweed extracts in the future, further research and extension work, such as that described below, is critical.

## New Australian seaweed extract research

### Chemical characterisation of seaweed extracts

The composition of liquid seaweed extracts is complex. In part, plant growth regulators have been used to explain the benefits of liquid seaweed extracts.

New research has extended the set of growth stimulant molecules found in seaweed extracts. Firstly, it is emerging that brassinosteroids are present in the Kelpak™ *E. maxima* seaweed extract (Stirk et al 2014). Secondly, in addition to brassinosteroids, strigolactones have been found in the Seasol™ seaweed extract (Arioli, unpublished data). However, another recent Australian report by Yusuf et al. (2012) that highlighted the complexity of seaweed extracts makes it difficult to ascribe the plant responses to a single growth stimulant.

Brassinosteroids and strigolactones have attributes that are related to some of the types of benefits found when seaweed extracts are applied to specific plants. The brassinosteroids have roles in flowering, plant structure and stress tolerance (Divi and Krishna 2009) and also have a recently discovered role in the innate plant immune system (Belkhadir et al 2012). The original role attributed to strigolactones was to stimulate seed germination of certain parasitic plants. However, more recent research defined strigolactones as having a role as a plant stress regulator in drought, salinity and nutrient responses (Marzec et al 2013; Ha et al 2014). From an agronomic perspective, the exogenous application of these plant stimulants increased plant productivity (Divi and Krishna 2009; Hayat et al 2012; Ha et al 2014). The identification of new plant growth molecules in different seaweed extracts emphasises the diverse composition of the various extracts and the complexity of their modes of action.

### The effect of a seaweed extract on seedling establishment of broccoli

In Australia, broccoli (*Brassica oleracea*) production requires high rates of nitrogen fertilisers at transplanting to stimulate the early growth of seedlings and high yields (Bakker et al 2009; Dimsey 2009). These inputs make broccoli production prone to nitrogen losses through leaching and volatilisation and can have detrimental impacts on the environment (Porter et al 2012). These factors are driving the consideration of alternative methods for stimulating early growth in broccoli.

In recent published research (Mattner et al 2013), two field trials were conducted in Victoria (Australia) to test the hypothesis that a seaweed extract stimulated broccoli establishment above standard fertiliser practices. The seaweed extract used is unique because it combines the plant benefits derived from two seaweed species in a single extraction process. The liquid seaweed extract is made by alkaline hydrolysis of *D. potatorum* and *A. nodosum* biomass, marketed as Seasol™. According to the label information, the extract is predominately *D. potatorum* and has a soluble solid level of 16 % (*w*/*w*). The trials were conducted on contrasting soil types, i.e. a clay-loam sodosol and a sandy podosol. Plant seedlings were soaked overnight in the extract (1:200 dilution) and, once planted, received three applications (days 0, 10, 20) through plant establishment, at rates of 2.5 L ha^−1^ and 25 L ha^−1^.

In the sodosol soil, the extract significantly increased the leaf number, stem diameter and leaf area of broccoli seedlings, as compared to controls by 6, 10 and 9 %, respectively, (*p* ≤ 0.05), irrespective of the application rate. The effect of the extract was less pronounced in broccoli grown in the sandy podosol soil type, but still increased seedling leaf area significantly (11 %, *p* ≤ 0.05) when applied at the highest application rate.

The study demonstrated that an extract from *D. potatorum* and *A. nodosum* had the capacity to improve the establishment of broccoli seedlings in an Australian farm setting without increased nitrogen. In addition, the findings suggested that the differences in cation exchange capacity, organic matter and/or leaching properties contributed to the variation in growth response to the extract between broccoli in the different soil types.

### The suppressive effect of a seaweed extract on infection of broccoli by *Plasmodiophora brassicae*

Clubroot caused by *Plasmodiophora brassicae* is considered the most important soil-borne disease of brassica crops (Donald and Porter 2009). Infection consists of two major biological stages, a primary phase that occurs in the root hair and a secondary phase that occurs in the cortex (Donald et al 2008). The secondary phase results in the formation of root galls that are characteristic of the disease.

Wite et al. (2015) provided a new understanding regarding the suppression of clubroot infection using a broccoli sand-culture technique to test the hypothesis that a liquid seaweed extract could reduce primary and secondary infection of broccoli by *P. brassicae*. A commercially available seaweed extract (Seasol™), with a soluble solids level of 16 % (*w*/*w*), was used in the study. The liquid seaweed extract is made using both *D. potatorum* and *A. nodosum* meal. Treatments included two dilutions of the extract in water (1:25 and 1:200), with distilled water as the control. At 14, 28, 45 days after inoculation, the researchers stained the roots with FFA phloxine B to observe the development of the infection by *P. brassicae*.

**Fig. 4:**
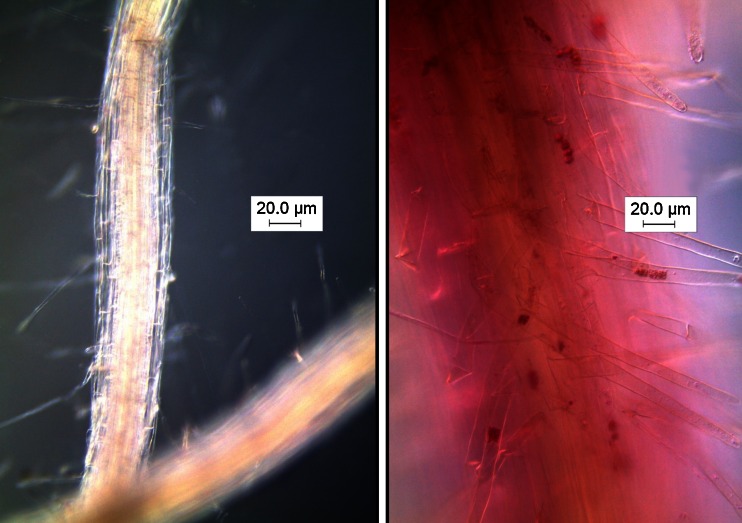
Zoosporangia (*stained red*) of *Plasmodiophora brassicae* in the root hairs of broccoli after 28 days. *Left panel*: broccoli roots treated with water. *Right panel*: broccoli roots treated with liquid seaweed extract (Seasol™)

After 45 days, the seaweed extract suppressed primary and secondary infection of broccoli by *P. brassicae* by up to 55 and 84 %, respectively, at both the dilutions (1:25 and 1:200) of the extract applied. Figure 4 demonstrates the ability of the seaweed extract to suppress the clubroot infection process. The underlying mechanism for this suppression is not known, but the authors suggested that the activation of natural plant resistance mechanisms and/or the presence of natural plant growth regulators may be involved. Importantly, this research is the first indication that a seaweed extract can directly suppress the damaging infection of broccoli by *P. brassicae*.

### The suppressive effect of a seaweed extract on *Sclerotinia minor*

*S. minor* is a devastating necrotrophic pathogen of a number of horticultural crops, including lettuce, green bean, cabbage, broccoli and others. Few published studies have considered the direct role of seaweed extracts in reducing the inoculum of fungal, soil-borne pathogens such as *S. minor*.

New published research (Mattner et al 2014) used a series of in vitro bioassays to test the hypothesis that a seaweed extract (Seasol™) made from both *D. potatorum* and *A. nodosum* has the potential to directly suppress the growth of mycelia and sclerotia of *S. minor* and reduce its ability to cause disease in lettuce. Treatments included the undiluted and diluted (1:500) extract and water controls buffered to the same pH as the liquid seaweed extract.

The study found that the undiluted extract suppressed the growth of *S. minor* by 18–100 %, depending on the bioassay method used. Inundation with the undiluted and diluted extract significantly suppressed the growth of sclerotia by 90 and 30 %, respectively, and reduced disease severity in lettuce seedlings. By comparison, controls buffered to the same pH as the extracts suppressed the growth of sclerotia by only 22 and 0 %, respectively. The report concluded that the seaweed extract comprising *D. potatorum* and *A. nodosum* had the potential to directly suppress the growth of *S. minor* and reduce its ability to cause disease. This effect is only partially due to the alkaline pH of the undiluted extract and other organic compounds were a likely main mode of action.

### The suppressive effect of a seaweed extract on *Albugo candida*

Seaweed extract was found to significantly reduce the incidence of white blister disease on broccoli leaves. White blister is caused by the fungal pathogen *A. candida*. White blister control is important because it can render produce unsaleable.

An Australian study regarding the reduction of *A. candida* was recently published (Mattner et al 2013). Symptoms of white blister disease were observed on leaves of broccoli at one of the broccoli plant establishment field trials reported by Mattner et al (2013). The field trial site that monitored for white blister was established as a randomised complete block design with four blocks. Treatments consisted of seaweed extract (Seasol™, soluble solids 16 % *w*/*w*) at 25 and 2.5 L ha^−1^ or water at 25 L ha^−1^. Commercial broccoli seedlings were soaked in either the seaweed extract (1/200 dilution in water) or water control overnight, transplanted into the field and received three drench applications of the diluted extract applied at 0-, 10- and 20-day time points. The researchers compared the number of infected leaves (those containing ≥1 pustule) per plant. They found a significant reduction by 23 % (*p* ≤ 0.05) in the incidence of white blister during the establishment stage in the field trial. This reduction occurred irrespective of the rate of the seaweed extract applied (2.5 or 25 L ha^−1^). The mode of action for this reduction is unknown but warrants further investigation. This is particularly the case for Victoria (Australia) where production areas of broccoli are affected by white blister.

The relevance of this work is high as it is the first report of a seaweed extract reducing the incidence of this commercially important pathogen. This approach warrants further testing since in other vegetables, liquid seaweed extracts of *A. nodosum* have been reported to reduce disease severity caused by foliar pathogens *Alternaria cucumerinium*, *Alternaria radicina*, *Didymella applanata* and *Botrytis cinerea* in carrot and cucumber (Jayaraj et al. 2008; Jayaraman et al. 2011).

### The phenotyping of seaweed extract efficacy

To understand the beneficial attributes of seaweed extracts on plants new labour-saving and smart technologies are being applied in testing systems (Rayorath et al 2008). Such systems should be automated, capture biological observations (such as plant phenotypes) and enable systematic interrogation of relevant data to produce meaningful scientific conclusions. The field of phenomics is based on such parameters. This is a dynamic and advancing new field (Summerer et al 2013; Brown et al 2014). The phenotyping of various seaweed extracts for their optimal application rates, their timing of application and genetic variation in plant responses are new opportunities.

In this regard, new insights from emerging research were presented as part of an oral presentation at the 5^th^ Congress of the International Society for Applied Phycology. A new prototype seaweed extract phenotyping system was described. It comprises of a low through-put plant-liquid assay system with a high-resolution time-lapse photography. Initially, the system was built to dynamically and visually phenotype plant roots grown in liquid seaweed extract. Subsequently, root growth was measured to determine root growth profiles specific for the seaweed extract. The system is still under development but provides an insight into a new way to compare and characterise the phenotypes of different seaweed extracts.

As an example of the system, growth was measured by comparing a series of high-resolution time-lapse photographs for either root or shoot growth. Root images for analysis were screened to avoid any roots that were tangled or overlapped, as this would compromise the ability to track root growth across the duration of the growth experiment. Once a root was selected, the point of the root initiation was marked and comparative growth rates were collected based on 24-h intervals. Daily root length was determined by overlapping the time-lapse images and the additional root length measured. A similar approach was used for shoot growth. A final summary of growth parameters is overlayed into the final time-lapse image, as a reporting dashboard.

This approach was also designed as a tool for the discovery of bioactives in seaweed extracts. It is a powerful assay because it enables the direct comparison of growth parameters based on changes in seaweed extract concentrations or composition versus a constant control.

**Fig. 5:**
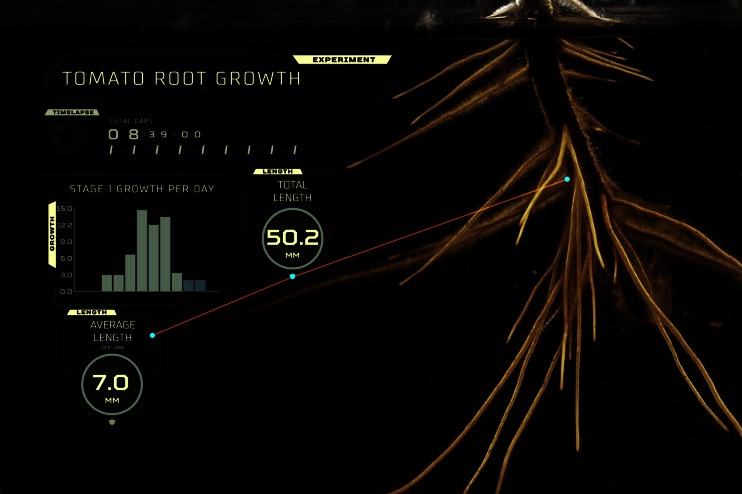
Time-lapse photo of tomato roots grown in liquid seaweed extract (Seasol™) after 8 days growth

Figure 5 demonstrates the use of the time-lapse photography system. The high-resolution image shows tomato root growth achieved after 8 days in a liquid seaweed extract. The lines mark the root that was tracked to measure growth rate across 10 days. The control was water since the purpose of the experiment was to visualise the effects of and eventually characterise bioactive molecules in the seaweed extract. The reporting dashboard in the image shows the root growth rates by day as a graph, the number of days the experiment was conducted and the average root length across the entire experiment.

Visualising the beneficial plant responses due to seaweed extracts is proving worthwhile. From these initial results, it is envisaged that the high-resolution phenomics approach will significantly advance our knowledge of seaweed extract application dosages, timing and plant development stages to achieve maximum crop productivity gain. This type of research is an important step towards enabling Australian farmers to capture the full benefits of applying seaweed extracts as part of an integrated agronomic programme.

## Conclusion

The liquid seaweed extract industry in Australia has a relatively long history, and this has enabled farmers to make productive use of seaweed extracts in agriculture and home gardeners to improve their gardens. Findings of this review reveal benefits to agriculture that include the improved establishment of crops such as *Brassica* spp. and resistance to specific and major diseases such as *Albugo candida* and *Sclerotinis minor*. This is consistent with the overseas scientific literature and on farm results. The active components in the treatments are however not yet understood, but the identification of potentially active components or synergies of components is emerging. Some of these include growth promoters such as brassinosteroids and strigolactones, but it is still unclear as to whether these or a synergy of molecules are effective, and bioassay-guided fractionation research will slowly reveal some of these complexities. To achieve these modern techniques that embrace smart and digital technologies are emerging, such as visual tools for phenotyping. This science extends a strong but relatively limited number of research papers published in Australia on seaweed extracts use in agriculture and contributes to a wider body of research globally.

The interest in seaweed extracts to enhance agricultural productivity continues to grow, both in Australia and globally. This is especially the case due to increasing crop productivity targets. The demand for food is increasing, but the supply of food is compromised by climate volatility and extreme weather patterns.

Seaweed extracts are complex and diverse in nature yet have great potential for enhancing crop productivity and offer novel biological mechanisms to exploit. In this regard, new research using advanced methodologies is critical and so is the translation of this new knowledge into practical outcomes that ensure farmers are able to adapt, innovate and survive. In addition, the diversity might be an opportunity to capture synergy from different seaweed extract combinations. In this regard, it is worth noting that overseas research uses seaweed extracts manufactured from a single source of seaweed, predominately *A. nodosum* or *E. maxima*, while the reports herein have focused upon a seaweed extract that is made from both *D. potatorum* and *A. nodosum*.

Scientific research of seaweed extracts has been building towards ultimately resolving their biological mechanisms and uncoupling the relationships between cause and effect.

However, for Australian agriculture to fully benefit from the use of seaweed extracts and to improve productivity, more scientific research, innovation and local field trials are needed. Agronomists, with expert knowledge of seaweed extracts, will have a fundamental role in enabling Australian farming operations to achieve both optimal productivity and economic benefits by routinely implementing seaweed extracts.
